# Identification of lower limb muscle fatigue in basketball players based on sEMG signals

**DOI:** 10.3389/fphys.2025.1689324

**Published:** 2025-10-02

**Authors:** Xiao Ma, Siwei Chen, Qiwei Li

**Affiliations:** ^1^ Sports Department, Shenyang Aerospace University, Shenyang, China; ^2^ School of Mechanical Engineering and Automation, Northeastern University, Shenyang, Liaoning, China

**Keywords:** basketball players, classification, muscle fatigue, sEMG, transformer

## Abstract

Muscle fatigue is an inevitable physiological phenomenon during exercise, which not only leads to a decline in athletic performance but also increases the risk of sports injuries. Therefore, effectively identifying an athlete’s muscle fatigue states is of critical importance. This study used the Transformer model to investigate the identification of lower limb muscle fatigue states in basketball players based on surface electromyography (sEMG) signals. The lower limb sEMG signals of 15 basketball players were collected during the experimental process, and the three muscles with higher contribution were selected by combining the muscle synergy analysis method, and then 8 types of feature signals were extracted and fused. The results showed that the Transformer fatigue recognition model based on fused features outperformed the single-feature model in all evaluation metrics. The classification accuracies of the three muscles were 94.28% ± 3.25%, 93.36% ± 3.87% and 94.11% ± 3.28% under the fusion-feature-based condition, respectively. In this paper, LSTM and XGBoost were selected as the comparison models, and the results showed that Transformer significantly outperforms the comparison models in all evaluation metrics, exhibiting stronger robustness and generalization ability.

## 1 Introduction

In sports, athletes frequently perform high-intensity movements such as jumping, accelerating, and changing direction, placing extremely high demands on neuromuscular control of the lower limb muscle groups (Taylor et al., 2015; Delextrat et al., 2015). Muscle fatigue, as an inevitable physiological phenomenon during exercise, not only leads to a decline in athletic performance but also significantly increases the risk of sports injuries (Abd-Elfattah et al., 2015; Bigland-Ritchie and Woods, 1984). Especially when muscles hav, e not yet shown obvious signs of fatigue, the absence of effective methods to identify muscle fatigue states can easily result in irreversible damage. Therefore, developing an accurate method for identifying lower limb muscle fatigue is of great significance for enhancing the scientific rigor and safety of athletic training (Castiblanco et al., 2021).

Currently, in the field of muscle fatigue identification, many researchers prefer to use multi-modal sensor technology to analyze athletic performance and fatigue states from multiple dimensions. Al-Quraishi et al. (2021) identified fatigue states during ankle joint motion using sEMG signals and electroencephalogram (EEG) signals. Mu et al. (2022) identified fatigue states in the biceps brachii during exercise using sEMG signals and electrocardiography (ECG) signals. Li and Chen (2022) identified fatigue states during rehabilitation training using sEMG signals and ECG signals. Wang et al. (2024) used wearable smart fabric armband strain sensor to detect changes in biceps thickness, and combined sEMG signals to identify biceps brachii fatigue states. While these methods indeed provide richer movement and physiological information under experimental conditions, they also have significant limitations: complex system deployment, high costs, and challenging data synchronization. These drawbacks hinder their long-term application in real-world exercise training and competition environments, especially under complex condition.

In contrast, sEMG sensors, as a non-invasive, portable, and real-time physiological signal acquisition method, offer significant advantages in monitoring movement states (González-Izal et al., 2012). The sEMG signals are electrical signals accompanying muscle contraction and provide a reliable representation of fatigue states (Stashuk, 2001; Merletti and Farina, 2016). They not only directly reflect changes in muscle excitation levels and movement load but also enable continuous acquisition of effective signals with minimal interference. Therefore, this study selects sEMG signals as the sole input information source (Karthick et al., 2018). This approach aims to reduce system complexity, enhance wearability and practicality, while exploring the possibility of achieving high-performance fatigue recognition under single-sensor conditions (Sonmezo et al., 2021a; Jebelli and Lee, 2019), (Sonmezo et al., 2021b).

To overcome the limitation of reduced information dimensionality inherent in single sensor, this study extracts multi-dimensional feature information from sEMG signals and uses feature fusion techniques to improve the accuracy of muscle fatigue recognition (Wei et al., 2020). When muscle fatigue occurs, sEMG signals exhibit changes in the time domain, frequency domain, and nonlinear features (Castiblanco et al., 2021; Lyu et al., 2024; Pan H. et al., 2024; Terracina et al., 2019; Kogami et al., 2022). For example, as fatigue progresses, the amplitude of the sEMG signals typically increase, manifested as an increase in the root mean square (RMS) value. The spectrum shifts toward lower frequencies, leading to a decrease in the median frequency (MF) and root mean square frequency (RMSF). And the complexity of the signal decreases, resulting in the changes in nonlinear metrics such as sample entropy (SE). To fully capture these dynamic changes in features, this paper uses a sliding window approach to process sEMG signals and uses a feature fusion strategy to increase the richness of input information, thereby enhancing the model’s accuracy in identifying muscle fatigue states.

Additionally, considering that muscles do not work independently during movement but rather perform complex movements through muscle coordination (Santuz et al., 2017; Safavynia et al., 2011; Fan et al., 2025). Therefore, this paper introduces muscle synergy analysis to identify the dominant muscle groups involved in specific movement tasks (Yu et al., 2024). By using Non-negative Matrix Factorization (NMF) to decompose multi-channel sEMG signals, the muscles that contribute most significantly to movement are identified. Fatigue recognition models are then established for these muscles individually, thereby enhancing the fatigue recognition capability based on single-source sEMG signals.

To this end, this paper proposes a lower limb muscle fatigue recognition method based on the Transformer model. First, lower limb sEMG signals are collected from 15 basketball athletes during standard movement tasks. NMF is used for muscle synergy analysis to identify the dominant muscle groups during movement. Subsequently, multiple features are extracted from the synergistic muscle signals to construct a fused feature input. And the Transformer model is used to identify the fatigue states. Experimental results indicate that the fused feature model outperforms single-feature models in terms of accuracy and robustness, effectively achieving fatigue state identification. And in this paper, Long Short-Term Memory (LSTM) and extreme Gradient Boosting (XGBoost) are selected as the comparison models, and the results show that Transformer significantly outperforms the comparison models in all evaluation metrics, exhibiting stronger robustness and generalization ability.

## 2 Experiments and methods

**FIGURE 1:**
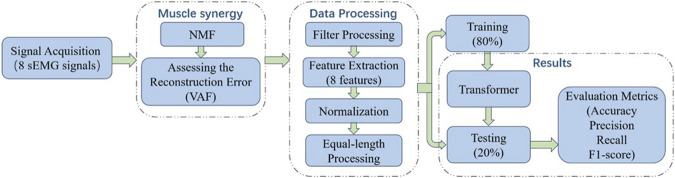
The workflow of signal acquisition and processing.

In the study of muscle fatigue identification using sEMG signals, signal acquisition and processing are a crucial step that have a significant impact on subsequent model training. Figure 1 illustrates the workflow of signal acquisition and processing.

### 2.1 Experiments and signal acquisition

This study collected data from 15 basketball athletes, including 10 males and five females. The participants’ ages ranged from 18 to 30 years, heights from 175 to 200 cm, and weights from 65 to 110 kg. All participants voluntarily participated in this study and were informed of the purpose, protocol, methods, and data collected in this trial. None of the participants had any pathological conditions or history of orthopedic surgery that could affect lower limb biomechanics.

**FIGURE 2:**
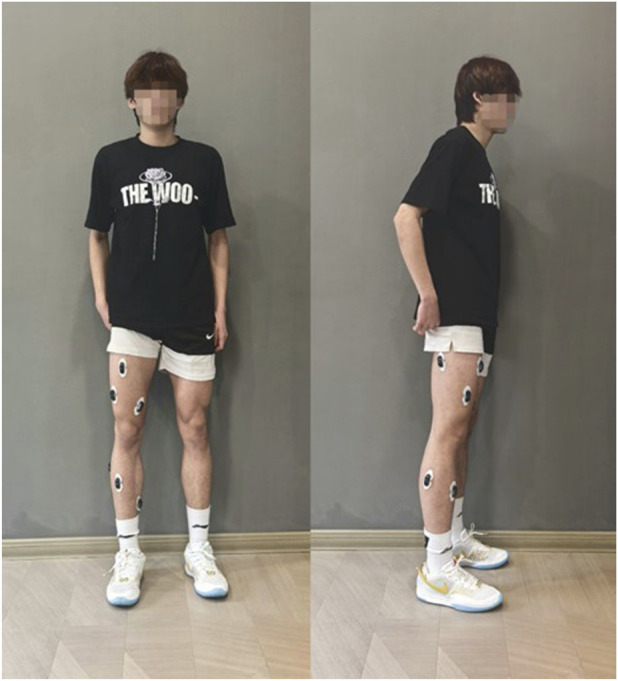
The participant wears Picos.

Each participant performed standard squat exercises to the point of fatigue after a warm-up session. The experiment was stopped when the participant could no longer perform squats. Each participant completed two sets of squats, with a rest period of 15–20 min between sets. To ensure consistent squat depth and speed, a metronome was used to guide the participants, and participants performed squats at a frequency of 40 repetitions per minute. Finally, invalid data due to connection issues or non-compliance with testing procedures were excluded. During the experiment, sEMG signals from 8 lower limb muscles were collected using the wireless sEMG sensor Pico (Cometa, Italy) at a sampling frequency of 2000 Hz. The sensor is a CE-approved medical-grade device with factory calibration completed by the manufacturer. When using the sEMG sensors, the skin was disinfected with alcohol and hair was trimmed appropriately to ensure proper adhesion of the sensors to the skin. The sEMG sensors were positioned parallel to the muscle fibers. Before the acquisition experiments participant wore the sensors for warm-up exercises to fully activate the sensors. And the participant wore Picos as show in Figure 2. The muscles and their corresponding numbers are listed in Table 1.

**TABLE 1:** Muscle names and numbers.

Muscles	Number	Muscles	Number
Vastus Medialis	1	Tibialis Anterior	5
Vastus Lateralis	2	Soleus	6
Rectus Femoris	3	Lateral Gastrocnemius	7
Biceps Femoris	4	Medial Gastrocnemius	8

### 2.2 Muscle synergy

During human movement, no movement is executed solely by an individual muscle; rather, it results from the coordinated activation of multiple muscles acting synergistically (Zhang et al., 2024; Turpin et al., 2021; W et al., 2025). During the experiments in this study, not all muscles reached the state of fatigue. In particular, muscles involved in non-dominant movements were less prone to fatigue. Therefore, this study used NMF to perform muscle synergy analysis on multi-channel sEMG signals, aiming to identify the dominant muscles involved in the movement and to achieve optimal muscle channel selection. Muscle synergy analysis is a typical blind source separation problem. NMF (Pan Z. et al., 2024), (W et al., 2025) achieves dimension reduction by decomposing a non-negative matrix into the product of two non-negative matrices, as shown in Equation 1.

$$V_{m\times n}=W_{m\times k}\times H_{k\times n}+E_{m\times n}$$ (1)

In the equation, V denotes the data matrix. W denotes the basis matrix. H denotes the coefficient matrix, which is obtained by reducing the dimension of matrix V through muscle synergy analysis. E denotes the error matrix, which is the difference between the reconstructed matrix from the two decomposed matrices and the original matrix. *m* denotes the number of samples. *n* denotes the number of sEMG signals collected, which is equal to 8, as the dataset contains sEMG signals from 8 muscles. *k* denotes the number of sEMG signals after muscle synergy analysis. When using NMF for muscle synergy analysis, it is essential to determine a reasonable value for *k* by evaluating the decomposition performance. The Variance Accounted For (VAF) is a commonly used metric for assessing the reconstruction error of NMF and is used to assess the fit between the reconstructed matrix and the original matrix (Chen et al., 2018).

### 2.3 Data processing

#### 2.3.1 Filter processing

Data processing is crucial in muscle fatigue recognition based on sEMG signals. In this paper, sEMG signals were filtered using a Butterworth bandpass filter to eliminate high-frequency noise and irrelevant signals, with the filter frequency range of 20–150 Hz. Additionally, a 50 Hz notch filter was used to eliminate interference from power frequency signals.

#### 2.3.2 Feature extraction

Data processing is crucial in muscle fatigue recognition based on sEMG signals. In this paper, sEMG signals were filtered using a Butterworth bandpass filter to eliminate high-frequency noise and irrelevant signals, with the filter frequency range of 20–150 Hz. Additionally, a 50 Hz notch filter was used to eliminate interference from power frequency signals. Muscle fatigue causes changes in sEMG amplitude, frequency, and signal complexity. Therefore, reasonable feature extraction plays a key role in accurately identifying muscle fatigue states. This paper extracted features from preprocessed sEMG signals in three aspects: time domain, frequency domain, and nonlinear features, in order to comprehensively capture the feature information of sEMG signals under fatigue states (Castiblanco et al., 2021; Lyu et al., 2024; Pan H. et al., 2024; Terracina et al., 2019; Kogami et al., 2022).

TD features directly reflect the intensity variation of signals over time and are among the most commonly used methods in sEMG signals analysis. In Equations 2–5, *X*_*i*_ represents the *i*th sample and *n* represents the total number of the samples.

RMS is a commonly used TD feature closely related to muscle contraction intensity, as shown in Equation 2. As fatigue increases, muscles require more motor units to maintain output force, resulting in a general increase in RMS values.

$$RMS=\sqrt{\frac{\sum_{i=1}^{n}X_{i}^{2}}{n}}\left(i=1,2,3...n\right)$$ (2)

IEMG represents the cumulative amplitude of the signals over a certain period of time, serving as a measure of the total muscle activity, as shown in Equation 3. As fatigue increases, muscle activation levels increase to maintain output, resulting in a general increase in IEMG values.

$$\text{IEMG}=\sum_{i=1}^{n}\left|X_{i}\right|$$ (3)

WL represents the amplitude variation within a specific window, providing a comprehensive indicator of signal complexity, as shown in Equation 4. Muscle fatigue may cause increased signal fluctuations, resulting in higher WL values.

$$WL=\sum_{i}^{n}\left|x_{i+1}-x_{i}\right|$$ (4)

SSC reflects rapid oscillations in the signal and acts as a counter for changes in signal slope, as shown in Equation 5. As fatigue increases, sEMG signals become smoother or exhibit specific patterns, and the value of SSC may decrease.

$$\text{SSC}=\sum_{i=3}^{n}\{\begin{array}{l} 1,if(\left(\text{sgn}\left(x_{i}-x_{i-1}\right)-\text{sgn}\left(x_{i-1}-x_{i-2}\right)\right)<0\\ 0,otherwise \end{array}\;\left(i=3,4,...,n\right)$$ (5)

FD features reveal the changes in the frequency composition of sEMG signals during muscle fatigue through power spectral analysis. These features are particularly effective for detecting the fatigue-induced spectral“left shift” phenomena. In Equations 6,7, [Disp-formula e7], *f*_*i*_ represents the *i*th frequency components of the input sEMG signals, *P*_*i*_ represents the power spectral values for the *i*th set of the input sEMG signals, *m* represents the total number of frequency bins or components being considered.

MF is the weighted average frequency of the signal’s power spectrum, as shown in Equation 6. And it is an important spectral indicator for fatigue assessment. As fatigue increases, the conduction velocity of muscle fibers decreases, causing MF to shift toward lower frequencies.

$$\text{MF}=\frac{\sum_{i}^{m}P_{i}}{m}\;\left(i=1,2,...,m\right)$$ (6)

RMSF reflects the overall trend of energy concentration in the frequency distribution, as shown in Equation 7. And it is an important feature in FD analysis. As fatigue increases, the high-frequency components decrease, resulting in a typical downward trend in RMSF value.

$$\text{RMSF}=\sqrt{\frac{\sum_{i}^{m}{f_{i}}^{2}\cdot P_{i}}{\sum_{i}^{m}P_{i}}}\;\left(i=1,2,...,m\right)$$ (7)

sEMG signals exhibit nonlinear and non-stationary characteristics. Therefore, extracting their complex dynamic features using nonlinear indicators can enhance the sensitivity and robustness of fatigue state recognition. In Equation 8, *N* represents the length of the original time series, *m* represents the length of compared sequences, *r* represents the tolerance threshold, and *B* ^*(m)*^ represents the average number of template vector pairs of length *m* whose distance is less than or equal to *r*. In Equation 9, *k* represents the scale factor (interval between points in the sub-series), and *L(k)* represents the average curve length across all sub-series at scale *k*.

SE reflects the complexity and uncertainty of a signal, with lower values indicating greater regularity, as shown in Equation 8. Under fatigue state, increased repetitiveness in neural drive patterns reduces the randomness of sEMG signals, resulting in the decrease in SE value.

$$SE\left(m,r,N\right)=-\ln\left(\frac{B_{m+1}}{B_{m}}\right)$$ (8)

FD reflects the geometric complexity of a signal and can reveal changes in neuromuscular regulatory mechanisms under fatigue state, as shown in Equation 9. Muscle fatigue may reduce the coordination of motor units, thereby affecting FD value.

$$FD=-\frac{d\log\left(L\left(k\right)\right)}{d\log\left(1/k\right)}$$ (9)

Since sEMG signals exhibit non-stationarity and time-varying characteristics, directly extracting features from the entire signal segment may obscure local dynamic information and fail to reflect real-time changes in muscle states. Therefore, this study employed the sliding window method for feature extraction of sEMG signals, with the window length of 100 m and the sliding step of 25 m.

#### 2.3.3 Normalization

Due to the large differences in the numerical ranges of various features, directly inputting them into a classification model can easily lead to training bias. Therefore, after feature extraction, each feature was normalized to eliminate the influence of scale discrepancies, thereby improving the model’s convergence speed and classification performance. This study used the Min-Max normalization method to map all feature values to the [0, one] interval, as shown in Equation 10. Specifically, for each experimental trial, the minimum and maximum values of each feature within that trial were used for normalization, where x represents the original feature value, where *x* represents the original feature value, *x*_min_ and *x*_max_ represent the minimum and maximum values of the feature in the training set, respectively, and *x’* represents the normalized value.

$$x^{\prime }=\frac{x-x_{\min}}{x_{\max}-x_{\min}}$$ (10)

#### 2.3.4 Equal-length processing

To avoid model performance degradation caused by inconsistent input data dimensions, equal-length processing was used to the normalized data. This ensured that the classification model receives inputs of identical structure across different samples, thereby enabling more accurate and reliable classification results. If the number of samples exceeded the standard sample count, the excess data was proportionally removed; if the number was below the standard sample count, missing samples were proportionally added. The added samples had the same numerical values as the preceding sample to maintain data continuity and smoothness.

### 2.4 Model settings

**FIGURE 3:**
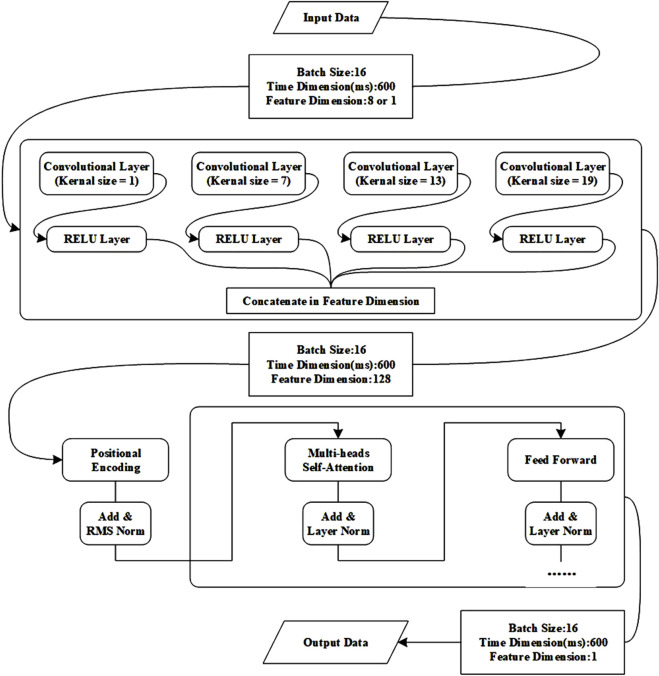
The process of Transformer.

To effectively capture the relationship between features in sEMG signals and fatigue states during movement, this paper used the Transformer model to identify athletes’ muscle fatigue states. The Transformer is a deep learning architecture originally proposed for natural language processing tasks, but its self-attention mechanism has demonstrated strong capabilities in modeling temporal dependencies in time-series data, including sEMG signals. In this study, we adopted the Transformer-based model to capture complex temporal and nonlinear patterns associated with muscle fatigue during movement (Vaswani, 2017). And Figure 3 shows the process of Transformer.

#### 2.4.1 Self-attention mechanism

At the core of the Transformer is the self-attention mechanism, which enables the model to dynamically weight the contribution of different time steps in the input sequence. Given a sequence of input features, the self-attention is computed as:

$$\text{Attention}\left(Q,K,V\right)=\text{softmax }\left(\frac{QK^{T}}{\sqrt{d_{k}}}\right)V$$ (11)

In the Equation 11, Q, K and V are the query, key, and value matrices obtained through learned linear transformations of the input, *d*_*k*_ is the dimension of the key vectors, and *T* is the time steps. This mechanism allows the model to focus on time points that are most informative for fatigue recognition.

#### 2.4.2 Multi-head attention and encoder design

To enhance the representation capability, the model incorporates multi-head attention, where multiple attention heads operate in parallel to capture diverse aspects of the temporal dependencies. The output of all heads is concatenated and passed through a linear transformation.

The Transformer encoder consists of several stacked identical layers, each containing: a multi-head self-attention sublayer, a position-wise feed-forward network with non-linear activation (typically ReLU), residual connections and layer normalization after each sublayer to stabilize training.

#### 2.4.3 Positional encoding

Since the Transformer architecture does not inherently encode sequence order, sinusoidal positional encoding is added to the input embeddings to provide temporal information. This allows the model to learn both relative and absolute temporal positions within the signal segments.

#### 2.4.4 Applicability to sEMG-Based fatigue recognition

By leveraging the global receptive field and dynamic weighting capability of self-attention, the Transformer effectively captures both short-term and long-term dependencies in sEMG data. This is particularly advantageous for identifying progressive patterns of muscle fatigue, which may not be evident in local time windows. Compared with traditional recurrent models, the Transformer allows for parallel computation and demonstrates superior performance on complex, high-dimensional physiological time-series data.

### 2.5 Evaluation metrics

In the task of muscle fatigue recognition, in order to comprehensively evaluate the performance of the established model, this paper selected a variety of classification evaluation metrics, including accuracy, precision, recall, and F1-score. These metrics can reflect the model’s performance in classifying fatigue and relaxed states from different perspectives.

Accuracy reflects the proportion of correctly classified samples to the total number of samples, as shown in Equation 12.

$$Acc=\frac{TP+TN}{TP+TN+FP+FN}$$ (12)

Precision reflects the proportion of true positive samples among all samples predicted as positive, as shown in Equation 13.

$$Pre=\frac{TP}{TP+FP}$$ (13)

Recall reflects the proportion of true positive samples that are correctly identified by the model among all actual positive samples, as shown in Equation 14.

$$Recall=\frac{TP}{TP+FN}$$ (14)

The F1-Score is the harmonic mean of Precision and Recall, as shown in Equation 15. It provides a balance between the two, especially in cases of imbalanced datasets.

$$F1-score=2\times \frac{Pre\times Recall}{Pre+Recall}$$ (15)

In Equations 12–15, *TP* denotes the number of true positive samples, *TN* denotes the number of true negative samples, *FP* denotes to the number of negative samples incorrectly predicted as positive, and *FN* denotes to the number of positive samples incorrectly predicted as negative.

## 3 Results

### 3.1 Muscle synergy results

**FIGURE 4:**
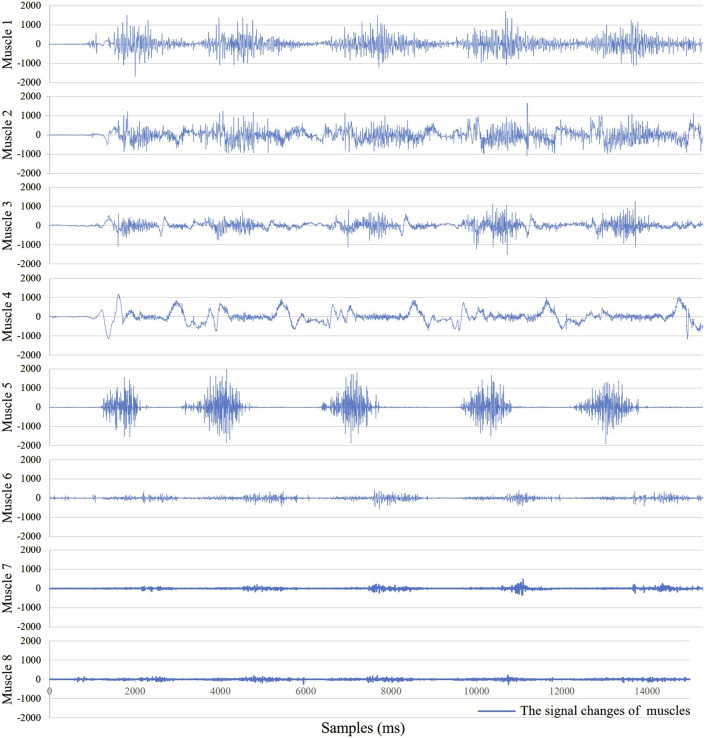
The sEMG signals of the 8 muscles.

**FIGURE 5:**
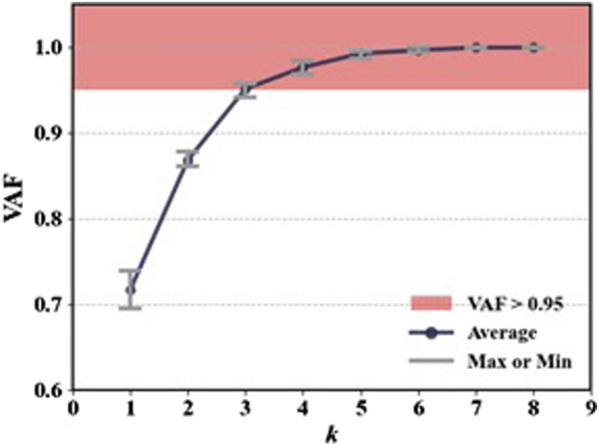
The VAF evaluation results.

As shown in Figure 4, for the same movement task, there are significant differences in signal amplitude among different muscles during the motion process. This suggests that not all muscles contribute equally in the movement. Therefore, it is necessary to select muscles highly correlated with the target movement. In this study, six squat movements were selected from each trial of every participant as input data for muscle synergy analysis. The evaluation was conducted using the VAF. Figure 5 shows the VAF evaluation results. The red areas in the figure represent regions where the average VAF value exceeds 0.95. As shown in Figure 5 and Table 2, the optimal number of muscle synergies is achieved when *k* = 3, and the VAF value is 0.951. Therefore, the number of muscles for muscle synergies is selected as 3.

**TABLE 2:** The VAF evaluation results.

*k*	1	2	3	4	5	6	7	8
Avg	0.717	0.868	0.951	0.956	0.992	0.996	0.999	0.999
Max	0.738	0.878	0.957	0.983	0.995	0.999	0.999	1
Min	0.695	0.86	0.941	0.968	0.987	0.993	0.998	0.998

**FIGURE 6:**
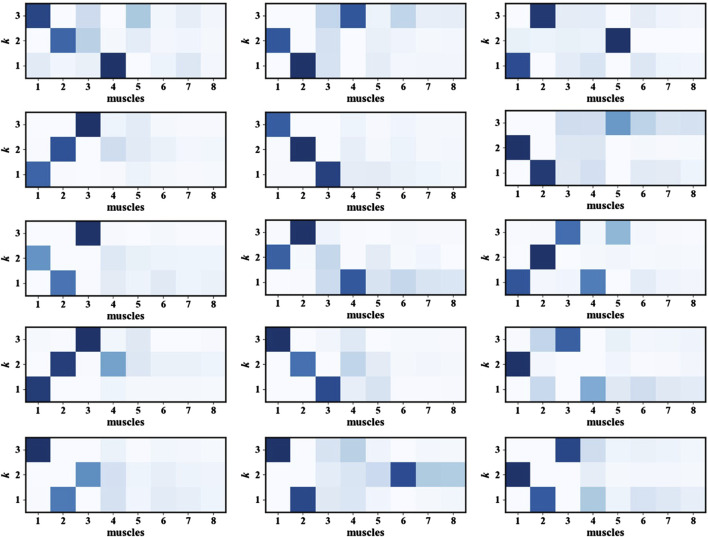
The heatmap of muscle synergy results.

After determining the number of muscle synergies *k*, the next step involved identifying the muscles most actively involved in the movement. This was achieved by analyzing the frequency of occurrence of each muscle channel across all synergies and selecting the most representative muscles accordingly for fatigue states recognition. Figure 6 presents the heatmap of muscle synergy results, and the intensity of the colors indicates the importance of the muscles during the movement. The muscle synergy analysis results show that the muscles with higher contribution degrees vary slightly among participants. These differences are likely due to individual differences in body structure, movement habits, or training experience. Despite individual differences, statistical analysis of the contribution degree distributions across all participants reveals that Muscles 1, 2, and three exhibit higher synergy contribution degrees in most participants. This indicates that these three muscles are more actively involved in this movement task and can provide more discriminative feature information. Therefore, in subsequent model training, selecting the sEMG signals of Muscles 1, 2, and 3 as model inputs can help highlight information most relevant to fatigue state recognition, thereby enhancing the model’s performance and robustness.

### 3.2 Classification results

**FIGURE 7:**
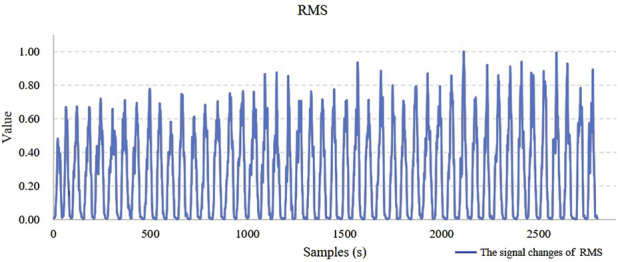
The trend chart of the RMS feature changes.

This paper extracted sEMG signals from three muscles (Muscle 1, 2, and 3) involved in the movement and extracted 8 types of feature information. These features exhibit regular changes during the process of muscle fatigue, but fatigue is a progressive process and the changes of a single feature before and after fatigue is not always pronounced. In some movements, substantial fluctuations may even occur. For example, in Figure 7, the trend chart showing how the RMS feature changes with fatigue levels, the overall RMS values tend to increase as fatigue progresses. However, in some movements, deviations from the average level may occur, and such fluctuations could affect the model’s classification accuracy. Therefore, relying solely on a single feature for fatigue state identification presents significant challenges.

**FIGURE 8:**
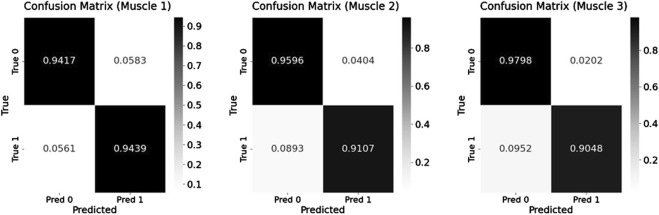
The confusion matrix results for fatigue states recognition under Equal-length processing.

**FIGURE 9:**
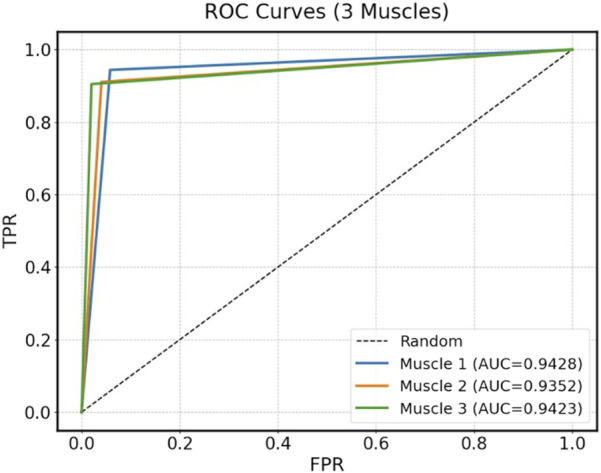
The ROC curve diagram.

To enhance recognition performance, this study used a multi-feature fusion approach to improve the model’s ability to distinguish muscle fatigue states. Specifically, this paper used the Transformer model to identify the fatigue states based on the sEMG signals from the three muscles identified through muscle synergy analysis. The model was trained using both the fused multi-feature data and the single-feature data as inputs, and the classification performance of the model under the two input conditions was compared. During model training, the dataset of each participant was divided into a training set and a test set. The training set was 80% and the test set was 20%, while 5-fold cross-validation was used to improve the generalization of the model. Table 3 presents the classification results under multi-feature and single-feature conditions, respectively. The results indicate that the classification results of the fused features outperform those of the single features across all evaluation metrics. Figure 8 shows the confusion matrix results for muscle fatigue states recognition under the fused feature condition. Figure 9 presents the ROC curve diagram.

**TABLE 3:** The classification results under multi-feature and single-feature conditions.

Muscle	Feature	Acc ± std (%)	Recall ± std (%)	Pre ± std (%)	f1_score ± std (%)
Muscle 1	RMS	88.89 ± 6.92	89.92 ± 8.64	89.78 ± 8.06	89.58 ± 6.61
	WL	80.14 ± 10.97	84.19 ± 13.21	75.89 ± 12.13	79.67 ± 12.07
	IEMG	87.33 ± 4.89	88.23 ± 5.86	86.91 ± 8.29	87.29 ± 4.81
	SSC	65.38 ± 14.26	85.2 ± 13.00	60.46 ± 18.09	69.95 ± 16.65
	RMSF	78.55 ± 8.72	82.05 ± 10.39	76.38 ± 12.31	78.26 ± 8.47
	MF	82.33 ± 8.11	85.42 ± 10.77	79.82 ± 13.9	81.59 ± 9.0
	SE	67.21 ± 8.67	73.15 ± 14.52	67.51 ± 13.03	69.1 ± 10.46
	FD	70.26 ± 10.45	78.63 ± 16.02	67.93 ± 15.57	71.6 ± 11.87
	Multi-Feature	94.28 ± 3.25	94.39 ± 3.15	94.39 ± 3.15	94.39 ± 3.27
Muscle 2	RMS	82.36 ± 5.95	81.49 ± 10.07	82.88 ± 6.78	81.77 ± 6.15
	WL	87.97 ± 8.67	87.6 ± 8.83	85.3 ± 12.12	86.22 ± 9.56
	IEMG	89.15 ± 7.07	89.62 ± 11.39	88.84 ± 7.43	88.86 ± 7.63
	SSC	72.15 ± 8.08	74.15 ± 9.07	73.42 ± 13.99	72.84 ± 7.52
	RMSF	78.67 ± 6.56	81.96 ± 12.3	75.79 ± 11.06	77.99 ± 9.11
	MF	77.07 ± 8.08	75.71 ± 13.2	79.44 ± 9.78	76.79 ± 8.74
	SE	65.92 ± 6.36	66.59 ± 13.53	67.04 ± 15.16	64.66 ± 7.69
	FD	73.74 ± 11.7	80.36 ± 11.81	69.82 ± 15.3	74.19 ± 12.57
	Multi-Feature	93.36 ± 3.87	91.07 ± 4.25	96.22 ± 2.45	93.57 ± 3.84
Muscle 3	RMS	86.53 ± 9.17	85.84 ± 15.23	84.52 ± 9.65	84.5 ± 10.8
	WL	86.79 ± 5.65	87.05 ± 10.73	88.5 ± 10.1	86.99 ± 6.15
	IEMG	89.0 ± 6.58	91.27 ± 11.61	89.03 ± 10.93	89.22 ± 7.07
	SSC	66.42 ± 11.38	41.39 ± 17.61	73.74 ± 26.61	52.33 ± 19.62
	RMSF	85.64 ± 7.98	87.66 ± 14.24	83.84 ± 9.53	85.05 ± 9.53
	MF	86.38 ± 10.48	89.17 ± 16.1	80.94 ± 12.88	84.57 ± 13.64
	SE	75.64 ± 6.57	62.42 ± 12.89	81.41 ± 14.97	69.63 ± 10.31
	FD	79.84 ± 11.47	75.78 ± 13.67	81.44 ± 11.47	78.38 ± 12.37
	Multi-Feature	94.11 ± 3.28	90.47 ± 4.95	97.93 ± 2.25	94.05 ± 3.37

**FIGURE 10:**
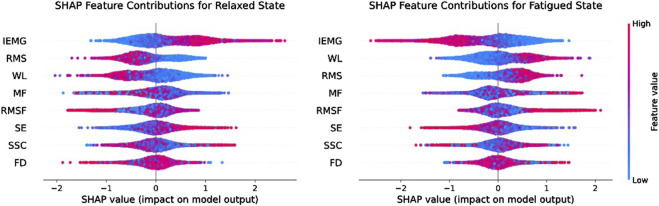
The results of SHAP analysis.

In order to further analyse the impact of individual features on the classification accuracy of the model, this paper used the SHapley Additive exPlanations (SHAP) analysis method during the model training process. This method can not only quantify the contribution of different features to the model classification results, but also present the importance ranking of different features in a visual way, and the results of SHAP analysis are shown in Figure 10.

**FIGURE 11:**
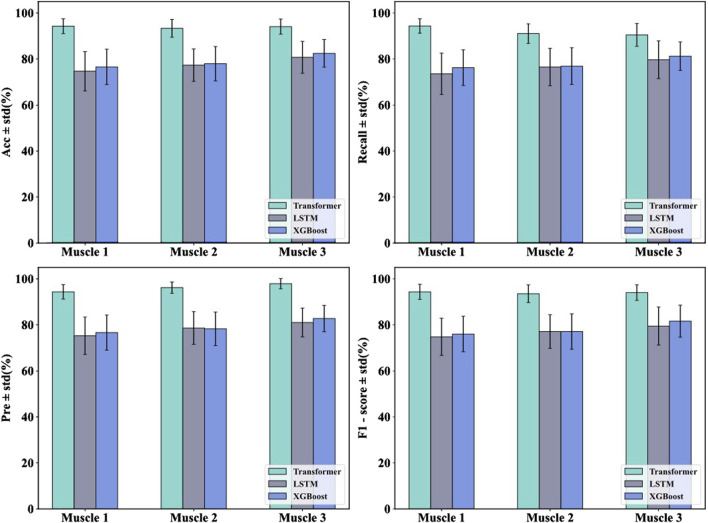
The classification results of the three models.

In order to verify the effectiveness of the Transformer model in the muscle fatigue recognition task, LSTM and XGBoost were selected as comparison models. By comparing with these two types of typical methods, the advantages of the Transformer model based on sEMG signals can be more comprehensively assessed. The results of the previous study showed that the feature fusion method was superior to any single feature, therefore, in the comparison experiments of the models, the fused features were used as inputs to the three models and the classification results were compared as shown in Table 4 and Figure 11.

**TABLE 4:** The classification results of the three models.

Muscle	Model	Acc ± std (%)	Recall ± std (%)	Pre ± std (%)	F1_score ± std (%)
Muscle 1	Transformer	94.28 ± 3.25	94.39 ± 3.15	94.39 ± 3.15	94.39 ± 3.27
	LSTM	74.71 ± 8.56	73.56 ± 9.00	75.29 ± 8.13	74.83 ± 8.07
	XGBoost	76.59 ± 7.70	76.26 ± 7.75	76.68 ± 7.63	76.03 ± 7.72
Muscle 2	Transformer	93.36 ± 3.87	91.07 ± 4.25	96.22 ± 2.45	93.57 ± 3.84
	LSTM	77.34 ± 7.05	76.53 ± 8.12	78.66 ± 7.12	77.13 ± 7.33
	XGBoost	77.90 ± 7.46	76.91 ± 7.99	78.33 ± 7.31	77.13 ± 7.70
Muscle 3	Transformer	94.11 ± 3.28	90.47 ± 4.95	97.93 ± 2.25	94.05 ± 3.37
	LSTM	80.76 ± 6.95	79.68 ± 8.20	81.04 ± 6.29	79.50 ± 8.28
	XGBoost	82.47 ± 6.00	81.26 ± 6.20	82.79 ± 5.75	81.64 ± 6.97

## 4 Discussion

Table 3 presents the average and standard deviation of classification accuracy, recall, precision, and F1-score for fatigue state recognition based on the Transformer model for three muscles under different feature inputs. It is evident that the multi-feature significantly outperforms any single feature across all metrics, demonstrating superior robustness and generalization capability in muscle fatigue states recognition. Some single features, such as IEMG and WL, also exhibit strong classification capabilities on certain muscles. However, by comparing the fatigue states recognition results of the three muscles under single features, it can be observed that different muscles exhibit varying sensitivities to certain features during the fatigue process. The fatigue states recognition performance of single features is more susceptible to influences such as muscle physiological structure, usage intensity, and signal noise, resulting in significant differences in classification performance of the same feature across different muscles. For example, the RMS feature achieves the accuracy of 88.89% on Muscle one but drops to 82.36% on Muscle 2. Similarly, the MF feature achieves the accuracy of 86.38% on Muscle 3, but drops to 77.07% on Muscle 2. To further explain the differences between features, this paper uses the SHAP analysis method to visualize and quantify feature importance. The results in Figure 10 show that the TD features (e.g., IEMG, RMS, and WL) exhibit high SHAP values in the muscle fatigue recognition task, indicating that they have a large contribution to the model classification decision, whereas the contribution of the nonlinear features (e.g., SE and FD) is relatively small, which is consistent with the classification performance of the single features in Table 3, and verifies the validity of SHAP for the interpretation of the model.

In contrast, the fused feature effectively integrates time-domain, frequency-domain, and nonlinear information, mitigating the instability or ineffectiveness of single features. Fused feature exhibits more stable performance across different muscles. The accuracy on Muscle 1, 2, and three are 94.28%, 93.36%, and 94.11%, respectively. In summary, single features have certain limitations in fatigue recognition, while fusing multi-feature information can significantly improve model performance, making it a more reliable and effective strategy for muscle fatigue states recognition. Therefore, muscle fatigue states recognition through feature fusion is highly necessary.

In order to verify the effectiveness of the proposed Transformer model in the muscle fatigue recognition task, LSTM and XGBoost are selected as the comparison models, and the comparison results are shown in Table 4 and Figure 11. Compared with LSTM and XGBoost, the Transformer model is able to capture both global and partial features, and integrate the TD, FD and nonlinear feature information through the mechanism of multi-head attention, so that it exhibits a more stable and superior classification performance in different muscles. After Friedman test and the *post hoc* Nemenyi test, it can be seen that Transformer is significantly higher than the comparison model in the evaluation metrics of accuracy, recall, precision and F1-score on all muscles (P < 0.05), showing stronger robustness and generalization ability.

As shown in Figure 8, the three muscles demonstrate high overall accuracy in the classification task, effectively distinguishing between relaxed and fatigued states. Among them, Muscle one demonstrates the most balanced classification performance, with an accuracy of 94.17% for the relaxed state and 94.39% for the fatigued state, indicating a nearly equivalent capability in recognizing both states. However, there are significant discrepancies in the classification accuracy of Muscle two and Muscle 3. The fatigue state recognition accuracy of Muscle two is 91.07%, significantly lower than relaxed state accuracy of 95.96%; the difference is even more pronounced in Muscle 3, with the relaxed state recognition accuracy of 97.98% and the fatigue state accuracy of only 90.48%. This result indicates that the model has certain limitations when identifying the fatigue state of specific muscles. Further analysis based on the muscle synergy heatmap shown in Figure 6 reveals that the muscles with higher contribution vary among participants. For all participants, the Muscle 1 has a high degree of contribution, so the difference in recognition between the relaxed and fatigue states of Muscle one is not significant. However, for some participants, the contribution of Muscle two and Muscle three is low, possibly indicating that these muscles did not fully reach the fatigue state during the experiment, leading to the decrease in the model’s ability to recognize the fatigue state.

These results suggest that although the overall model has good classification ability, it is still affected by differences in muscle activation patterns when generalized to all individuals. Therefore, in practical applications, it is necessary to construct personalized models based on the muscle synergy characteristics of different participants. Models pre-trained based on large-scale populations can be adapted to new individuals using a small amount of individual-specific data through transfer learning methods. And adaptive fine-tuning methods that iteratively update model parameters based on individual responses can further enhance personalization.

In this study, the sample size is relatively small (15 athletes), and although this sample size is sufficient as a preliminary study to validate the feasibility of the proposed method, the limited dataset may restrict the generalizability of the model. Therefore, in practical applications, it is necessary to collect a larger and more diverse group of subjects, while covering different types of exercise tasks, in order to further improve classification performance and model adaptability.

Furthermore, this study focuses on the squat movement because it is a standardized and repeatable motor pattern that can reliably induce fatigue under controlled conditions. However, the proposed method itself is not limited to this specific task. The fatigue identification framework can be extended to other exercise types by identifying highly engaged muscle groups through muscle synergy analysis and subsequently recognizing their fatigue states. the methodological framework remains consistent even if the dominant muscles involved vary across different movements (e.g., squatting, sprinting, cutting, or jumping). Therefore, this fatigue recognition framework can be extended to dynamic specialized exercise tasks to enhance the practical relevance in competitive scenarios.

## 5 Conclusion

This study used the Transformer model to recognize lower limb muscle fatigue states in basketball players based on sEMG signals. By collecting sEMG signals from the lower limbs of 15 basketball players and combining muscle synergy analysis methods, this paper selected key muscle groups with higher contribution during movement tasks, providing a more representative signal basis for features extraction.

In the experiment, this paper extracted and fused 8 types of time domain features, frequency domain features and nonlinear features, and compared their performance with a single-feature model. The results showed that the fused features significantly improving the model’s robustness and recognition accuracy, particularly demonstrating higher consistency and stability across different muscles. The Transformer fatigue recognition model with fused feature outperforms the single feature model in terms of accuracy, precision, recall, and F1-score, validating the effectiveness of the multi-feature fusion strategy. In this paper, LSTM and XGBoost were selected as the comparison models, and the results showed that Transformer significantly outperforms the comparison models in all evaluation metrics, exhibiting stronger robustness and generalization ability.

However, during the experiment, it was found that there were differences in muscle activation states among different participants, which may affect the recognition performance of the model.

Therefore, future research can further develop personalized models targeting muscle synergy characteristics of different participants and increase the number and diversity of participants to enhance classification performance and model adaptability. Moreover, constructing real-time fatigue recognition systems could enable broader applications in sports rehabilitation, ergonomic assessment, and human-machine interaction.

## Data Availability

The raw data supporting the conclusions of this article will be made available by the authors, without undue reservation.
